# Acetylcholinesterase Inhibitor Ameliorates Early Cardiometabolic Disorders in Fructose-Overloaded Rat Offspring

**DOI:** 10.3390/ph17081055

**Published:** 2024-08-10

**Authors:** Victor Hugo Martins de Miranda, Camila Paixão Dos Santos, Pietra Petrica Neves, Antonio Viana Nascimento-Filho, Marina Rascio Henriques Dutra, Nathalia Bernardes, Maria Claúdia Irigoyen, Kátia De Angelis

**Affiliations:** 1 Physiology Department, Federal University of Sao Paulo (UNIFESP), Sao Paulo 04023-062, Brazil; vhm.miranda@unifesp.br (V.H.M.d.M.); camilapaixao22@hotmail.com (C.P.D.S.); antonio.viana.nascimento@gmail.com (A.V.N.-F.); 2 Laboratory of Translational Physiology, Nove de Julho University (UNINOVE), Sao Paulo 01525-000, Brazil; pietrapetrica1@gmail.com (P.P.N.); marinarhdutra@gmail.com (M.R.H.D.); 3 Postgraduate Program in Physical Education, São Judas Tadeu University, Sao Paulo 03166-000, Brazil; nbernardes@outlook.br; 4 Hypertension Unit, Heart Institute (InCor), School of Medicine, University of Sao Paulo, Sao Paulo 05403-000, Brazil; hipirigoyen@gmail.com

**Keywords:** galantamine, fructose, offspring, heart rate variability, arterial pressure variability, arterial pressure, insulin resistance

## Abstract

Background: We investigate the role of galantamine on autonomic dysfunction associated with early cardiometabolic dysfunction in the offspring of fructose-overloaded rats. Methods: *Wistar* rats received fructose diluted in drinking water (10%) or water for 60 days prior to mating. Fructose overload was maintained until the end of lactation. The offspring (21 days after birth) of control and fructose-overloaded animals were divided into three groups: control (C), fructose (F) and fructose + galantamine (GAL). GAL (5 mg/kg) was administered orally until the offspring were 51 days old. Metabolic, hemodynamic and cardiovascular autonomic modulation were evaluated. Results: The F group showed decreased insulin tolerance (KITT) compared to the C and GAL groups. The F group, in comparison to the C group, had increased arterial blood pressure, heart rate and sympathovagal balance (LF/HF ratio) and a low-frequency band of systolic arterial pressure (LF-SAP). The GAL group, in comparison to the F group, showed increased vagally mediated RMSSD index, a high-frequency band (HF-PI) and decreased LF/HF ratio and variance in SAP (VAR-SAP) and LF-SAP. Correlations were found between HF-PI and KITT (r = 0.60), heart rate (r = −0.65) and MAP (r = −0.71). Conclusions: GAL treatment significantly improved cardiovascular autonomic modulation, which was associated with the amelioration of cardiometabolic dysfunction in offspring of parents exposed to chronic fructose consumption.

## 1. Introduction

Since the development of high fructose corn syrup (HFCS), fructose has become the main sweetener used by the food industry in processed foods, such as sugar-sweetened beverages. These beverages are considered the main source of consumption by adolescents and adults in many countries [1]. 

Clinical and experimental studies have shown that high fructose consumption is associated with many metabolic disorders of metabolic syndrome (MetS), such as hypertension, obesity, insulin resistance and dyslipidemia. MetS increases the risk of developing cardiometabolic diseases and is correlated with cardiovascular autonomic dysfunction [1,2]. Our group showed that rats exposed to 10% fructose (in drinking water) developed cardiovascular autonomic dysfunction and other metabolic disorders [3,4].

In this context, epidemiologic studies have shown that maternal high-sugar diets during pregnancy and lactation predispose offspring to develop metabolic disorders in adulthood. According to the Developmental Origins of Health and Disease (DOHaD) hypothesis, in utero and perinatal behavioral and environmental events increase the risk of developing metabolic disorders later in life [1]. Thus, many rodent studies have shown that high maternal fructose consumption during pregnancy and lactation increases the risk of developing cardiometabolic disease in offspring later in life [5]. 

Some clinical and experimental studies proposing alternative treatments for MetS showed significant results with the use of galantamine (GAL), a centrally acting acetylcholinesterase inhibitor approved for the treatment of Alzheimer’s disease [6]. GAL treatment improved cardiovascular autonomic dysfunction and some metabolic disorders and had a positive effect on neuroimmune dysfunction associated with MetS [7,8]. However, the role of GAL treatment on cardiovascular autonomic dysfunction and its effect on early cardiometabolic disorders observed in offspring of parents exposed to high fructose consumption remains unknown. Therefore, the aim of this study was to investigate the role of GAL on autonomic dysfunction associated with early cardiometabolic dysfunction in the offspring of fructose-overloaded rats.

## 2. Methods

### 2.1. Animals

*Wistar* rats were randomized into two groups at 21 days of age (35–55 g) and received fructose overload (D-fructose, 100 g/L) in drinking water or water (control group) until 60 days. After 60 days, the rats were bred until positive sperm were detected in their vaginal smears. The pregnant dams continued to receive the same diet (fructose or water) and were freely fed with standard laboratory chow and individually housed in plastic cages in temperature-controlled rooms (22 °C) with a 12:12 h dark/light cycle until lactation. One day after weaning (21 days after birth), the offspring (35–55 g) were divided into three groups (*n* = 6/3 females and 3 males): control group (C), fructose group (F) or fructose group treated with galantamine (GAL, Prati-Dunaduzzi, Pharmaceutical Industry, Jandira, Sao Paulo, Brazil) at a dose of 5 mg/kg/day. All groups received a standard diet and water without the addition of fructose, were individually housed in plastic cages in temperature-controlled rooms (22 °C) with a 12:12 h dark/light cycle and were evaluated 30 days after weaning (before the onset of puberty). For galantamine treatment, the fructose-fed parent offspring group received a dose of 5 mg/kg/day orally by gavage once a day for 4 weeks (30 days after weaning) to mimic the route of administration of this drug in humans [7]. All surgical procedures and protocols were approved by the Ethics Committee of the Federal University of Sao Paulo (protocol 4791091019) and were performed in accordance with the National Institutes of Health Guide for Care and Use of Laboratory Animals. 

### 2.2. Arterial Catheterization and Hemodynamic Assessments

On the last day of the protocol, all offspring were anesthetized, and a cannula was implanted in the carotid artery for direct measurement of arterial pressure (AP). Hemodynamic measurements were performed in conscious and awake rats in their cages at least 24 h after catheter placement. The arterial cannula was connected to a transducer (Blood Pressure XDCR, Kent Scientific, Torrington, CT, USA), and AP signals were recorded over a 30 min period using a microcomputer equipped with an analog-to-digital converter (Windaq, Dataq Instruments Inc., Akron, OH, USA, 2 kHz). The recorded data were analyzed on a beat-to-beat basis to quantify changes in systolic AP (SAP), diastolic AP (DAP), mean AP (MAP) and heart rate (HR) [9]. 

### 2.3. Autonomic Control of Heart Rate

For analysis of cardiovascular autonomic modulation in the time and frequency domains, the time series (three time series of 5 min for each animal) of the pulse interval (PI) and SAP were cubic spline-interpolated (250 Hz) and cubic spline-decimated to be equally spaced in time after linear trend removal; power spectral density was obtained by fast Fourier transform. The spectral power for low-frequency (LF-PI, 0.20–0.75 Hz) and high-frequency (HF-PI, 0.75–4.0 Hz) bands was calculated by power spectral density integration within each frequency bandwidth using a custom routine (Cardioseries Software, Daniel Penteado, Ribeirao Preto, Brazil). The time domain variable was the root mean square of the successive differences of the PI index (RMSSD), a representative index of vagal modulation [9]. Beat-to-beat values of SAP and PI intervals were used to estimate cardiac baroreflex sensitivity (BRS) by spectral analysis using the alpha index for the low-frequency band (0.20–0.75 Hz). Alpha index analysis evaluates short-term changes in systolic blood pressure and RR interval. This method has been proposed to quantify causal events related to the baroreflex [9].

### 2.4. Evaluation of Glycemia and Insulin Tolerance Test

On the last day of the protocol, the offspring were weighed, and blood glucose concentration was measured (Accu Check Roche, Sao Paulo, Brazil) after 4 h of fasting. The insulin tolerance test was performed to obtain the constant rate of blood glucose disappearance (KITT) as previously described [10].

### 2.5. Statistical Analysis

Data are expressed as mean ± standard error of the mean (SEM). Levene’s test was used to evaluate homogeneity. One-way ANOVA followed by a Student–Newman–Keuls test was used to compare groups. Pearson’s correlation was used to determine the association between variables. All data were analyzed using GraphPad Prism 8 software on Windows (GraphPad Software Inc., San Diego, CA, USA). The significance level was set at *p* < 0.05.

## 3. Results

In the present study, *Wistar* rats (genitors: three males and three females) were randomized into two groups at 21 days of age and received a fructose overload in drinking water or water. After 60 days, the males were mated with females until sperm were detected in their vaginal smears. The pregnant dams were then housed individually in plastic cages and continued to receive the same diet (fructose or water) until the end of lactation. The offspring of water-fed rats were assigned to the C group (three males and three females), and the offspring of fructose-fed rats were randomly assigned to the F and GAL groups (three males and three females/groups). 

**Figure 1:**
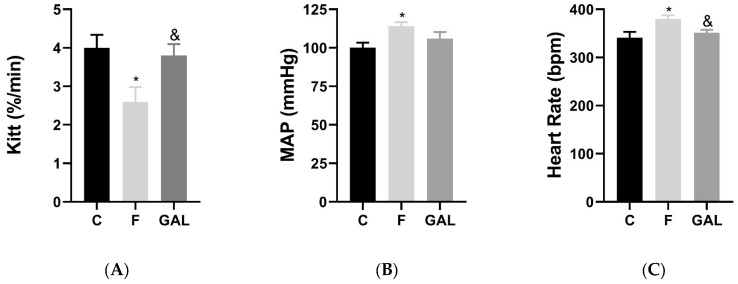
(**A**) Rate constant of glucose disappearance (KIIT). (**B**) Mean arterial pressure (mmHg). (**C**) Heart rate (bpm). * *p* < 0.05 vs. C; & *p* < 0.05 vs. F. Control (C, *n* = 6), fructose (F, *n* = 6) and fructose treated with galantamine (GAL, *n* = 6).

At the end of the protocol, there was no difference in body weight (C: 204 ± 26, F: 231 ± 26, GAL 198 ± 18 g) or blood glucose (C: 109 ± 4.78, F: 110 ± 3.48, GAL 111 ± 3.49 mg/dL) between groups. For the constant rate of blood glucose disappearance (KITT), we observed a decrease in the F group (2.60 ± 0.38%/min) compared to the C group (4 ± 0.34%/min) (Figure 1A). However, galantamine treatment (GAL: 3.80 ± 0.30%/min) showed an increase in this parameter compared to the F group (Figure 1A). 

In the hemodynamic parameters, we did not observe any significant difference between the groups (C, F and GAL) regarding DAP and SAP (Table 1). Parental fructose consumption caused an increase in MAP (Figure 1B) and HR in animals in the F group compared to the C group (Figure 1C). No difference in MAP was observed between the C and GAL-treated groups (Figure 1B). In addition, the GAL group showed a reduction in HR compared to animals in the F group (Figure 1C).

Regarding heart rate variability parameters, only drug-treated animals (GAL group) showed an increase in RMSSD and high-frequency band (HF–PI) compared to rats in groups C and F (Table 1). Regarding the low-frequency band (LF–PI), there was no significant difference among all groups (C, F and GAL). For the parameter related to cardiac sympathovagal balance (LF/HF), the F group had an increase compared to the C group. Treatment with GAL (GAL group) was effective in causing a reduction in this balance in the F group (Table 1).

In the vascular autonomic modulation, the F group showed an increase both in the variance in SAP (VAR–SAP) and in the low-frequency band of systolic arterial pressure (LF-SAP) compared to the C group. Treatment with GAL was effective in reducing VAR-SAP and LF-SAP. Moreover, the F + GAL group showed a significant increase in the alpha index compared with the F group (Table 1).

**Figure 2:**
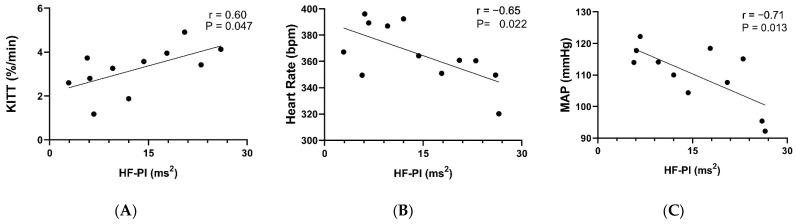
Pearson correlations involving offspring of fructose-fed and fructose + galantamine-fed rats. Positive correlation: (**A**) HF–PI and KITT. Negative correlations: (**B**) HF–PI and heart rate; and (**C**) HF–PI and MAP.

Pearson’s correlation analysis was performed to test the association between cardiac vagal autonomic modulation and cardiometabolic parameters in the fructose groups (F and F + GAL). A positive correlation was found between HF–PI and KITT (r = 0.60 *p* < 0.047 Figure 2A). Negative correlations were found between HF–PI and heart rate (r = −0.65 *p* < −0.022 Figure 2B) and MAP (r = −0.71 *p* < 0.013 Figure 2C).

## 4. Discussion

In the present study, we confirmed early cardiometabolic abnormalities in the offspring of fructose-overloaded rats. In addition, we observed impaired cardiovascular autonomic modulation in this condition. However, the main finding of the present study was the marked improvement in heart rate and arterial pressure variability associated with GAL treatment, which correlated with the attenuation of cardiometabolic dysfunction in the offspring of parents with a high-fructose diet.

In fact, the F group showed a lower insulin sensitivity (KITT) but without alteration in final body weight and blood glucose concentrations. In the context of insulin resistance, animal studies have shown that maternal high-fructose feeding promotes hyperinsulinemia in fetal and adult offspring [11]. The metabolites of maternal fructose hepatic metabolism, such as uric acid and free fatty acids, play important roles in mediating insulin resistance in systemic and local tissues [12]. However, the formation of an inflammatory response and oxidative stress from metabolites may also be involved in the development of insulin resistance in offspring [12,13].

Regarding body weight and blood glucose concentrations, our data showed that the consumption of 10% fructose (in drinking water) was not sufficient to change these parameters. This observation was different from other studies that showed an increase in body weight as one of the changes associated with fructose consumption. However, many of the experimental models use concentrations (50 and 60%) above the total caloric intake of fructose in humans, which is about 10% [12]. Our results are somewhat consistent with other studies that found metabolic changes in the offspring of parents exposed to 10% fructose (in drinking water) [14,15].

In addition, we observed changes in hemodynamic and autonomic parameters. Thus, the increase in sympathovagal balance (LF/HF ratio) demonstrates the prevalence of sympathetic modulation in the heart, which was followed by an increase in the variance in SAP (VAR-SAP) and sympathetic modulation of systolic arterial pressure (LF-SAP). Both indices may have contributed to changes in hemodynamic parameters, promoting an increase in MAP and HR. These changes are like those observed in other studies in animals that were overloaded with fructose [3]. Recently, in a study published by the laboratory group, the authors showed that autonomic dysfunction assessed by baroreflex sensitivity in the offspring of parents with a high-fructose diet was also correlated with reduced insulin sensitivity [16]. It is also known that the autonomic imbalance in MetS, characterized by increased sympathetic activity and reduced parasympathetic activity, favors insulin resistance [17]. In this context, we believe that autonomic changes may have contributed to the decrease in insulin sensitivity in the F-group animals.

In contrast to the untreated group of animals, the use of GAL improved insulin sensitivity. In fact, we have chosen the GAL dose of 5 mg/kg considering that several experimental models have shown beneficial effects on the inflammatory profile, oxidative stress and metabolic dysfunction [7,18]. In this case, we believe that this condition could be related to the increase in modulation of the parasympathetic nervous system caused by the effect of GAL. In autonomic parameters, we observed that GAL increased representative indices of cardiac parasympathetic modulation, such as RMMSD and HF–PI, and reduced sympathovagal balance (LF/HF ratio). This condition reduced the variance in systolic pressure and vascular sympathetic modulation, in addition to the normalization of MAP and a reduction in HR. In this context, the increase in cardiac parasympathetic modulation may be caused by an improvement in the ability of the alpha index, which indicates an increase in baroreflex control, to maintain MAP at constant pressure levels through rapid regulation of HR [19]. Our group has shown that fructose consumption in adult animals promotes damage to baroreflex function, which may be related to increased cardiac and vascular sympathetic modulation [3]. Santos et al. [16] argue that the baroreflex dysfunction observed in the F-group offspring in their study would be associated with increased sympathetic activity, which is directly related to increased AP [16]. Thus, there is a possibility that the predominance of parasympathetic modulation over sympathetic modulation due to the use of GAL may have resulted in beneficial effects on baroreflex activity in the drug-treated animals.

In experimental models of obesity and type 2 diabetes, the authors showed positive effects on metabolic parameters, such as insulin signaling, after the use of GAL. According to the authors, this improvement is related to the central effects of the drug that stimulate the vagal efferent pathways, which may have contributed to the reduction in the inflammatory profile, oxidative stress and other metabolic changes. In addition, there is the possibility of activation of the cholinergic anti-inflammatory pathway (CAP) by GAL. In these studies, the effect of GAL could be to modulate inflammation through increased activation of α7 nicotinic receptors (α7nAChR), which would reduce inflammation [7,20].

In our study model, we believe that these effects related to GAL may have ameliorated the fructose-induced metabolic alterations in the offspring. In this context, the increase in indices representing cardiac parasympathetic modulation may indicate that the increase in vagal activity in the central region by GAL may contribute to the improvement of metabolic abnormalities, as in insulin signaling. Correlational analyses confirm that the improvement in HF-PI with GAL treatment increased insulin sensitivity. This increase in cardiac parasympathetic modulation can be observed by the reduction in heart rate, which influences the decrease in MAP. Although we did not analyze this improvement in vagal signaling, it may have influenced the normalization of the inflammatory state through the activation of CAP by GAL. In a clinical study, the use of therapeutic doses of GAL in subjects with MetS showed results similar to ours. Subjects had reduced sympathovagal balance in the cardiovascular system and improved insulin sensitivity, followed by reduced inflammation and oxidative stress [8].

A limitation of this study is that the specific pathways by which GAL (galantamine) exerts its beneficial effects in the offspring of fructose-overloaded rats are not fully understood. Although our results demonstrate that GAL improves cardiac and vascular autonomic modulation, spontaneous baroreflex sensitivity and cardiometabolic parameters, the role of the CAP in these benefits, as well as its influence on inflammation and oxidative stress modulation, was not evaluated. Therefore, further research is needed to investigate the therapeutic potential of GAL in relation to the CAP, particularly regarding its ability to modulate inflammation and oxidative stress, two important mechanisms associated with the development and progression of disease across the lifespan. These additional studies will help to better explain the effects of parental fructose consumption on the development of cardiometabolic changes in their offspring.

In conclusion, treatment with GAL markedly improved heart rate and arterial pressure variability, which correlated with the attenuation of cardiometabolic dysfunction in the offspring of parents with a high-fructose diet. These findings suggest a beneficial role for therapies associated with neuroimmune modulation in the prevention of cardiometabolic disorders associated with the development of MetS.

## Figures and Tables

**Table 1:** Hemodynamic and autonomic evaluation in offspring groups.

	C	F	GAL
DAP (mmHg)	84 ± 3.4	95 ± 2.47	88 ± 3.8
SAP (mmHg)	117 ± 2.7	130 ± 3.5	121 ± 4.9
RMSSD (ms)	4.84 ± 0.73	4.67 ± 0.46	8.54 ± 0.52 *&
HF–PI (ms^2^)	7.65 ± 1.92	7.15 ± 1.30	21.41 ± 1.99 *&
LF–PI (ms^2^)	2.39 ± 0.67	3.15 ± 0.37	3.27 ± 0.27
LF/HF	0.33 ± 0.01	0.52 ± 0.10 *	0.17 ± 0.02 &
VAR–SAP (mmHg^2^)	13.80 ± 1.68	20.47 ± 2.38 *	11.51 ± 2.07 &
LF–SAP (mmHg^2^)	2.03 ± 0.48	4.13 ± 0.50 *	1.85 ± 0.39 &
Alpha LF index (ms/mmHg)	1.21 ± 0.13	0.85 ± 0.10	1.47 ± 0.14 &

Data are reported as mean ± SEM. Control (C, *n* = 6), fructose (F, *n* = 6) and fructose treated with galantamine (GAL, *n* = 6). DAP: diastolic arterial pressure, SAP: systolic arterial pressure, MAP: mean arterial pressure, RMSSD: root mean square of the successive differences. LF–PI: low frequency band, HF–PI: high frequency band, LF/HF: cardiac sympathovagal balance, VAR–SAP: variance in systolic arterial pressure, LF–SAP: low frequency component (vascular sympathetic) of systolic arterial pressure. * *p* < 0.05 vs. C; & *p* < 0.05 vs. F.

## Data Availability

The data that support the findings of this study are available from the corresponding author, K.D.A., upon reasonable request.
